# Towards an Interoperability Landscape for a National Research Data Infrastructure for Personal Health Data

**DOI:** 10.1038/s41597-024-03615-3

**Published:** 2024-07-13

**Authors:** Carina Nina Vorisek, Sophie Anne Inès Klopfenstein, Matthias Löbe, Carsten Oliver Schmidt, Paula Josephine Mayer, Martin Golebiewski, Sylvia Thun

**Affiliations:** 1 Berlin Institute of Health at Charité – Universitätsmedizin Berlin, Core Facility Digital Medicine and Interoperability, Charitéplatz 1, 10117 Berlin, Germany; 2 Charité – Universitätsmedizin Berlin, corporate member of Freie Universität Berlin and Humboldt-Universität zu Berlin, Institut für Medizinische Informatik, Charitéplatz 1, 10117 Berlin, Germany; 3 Institut für Medizinische Informatik, Statistik und Epidemiologie (IMISE), Universität Leipzig, Leipzig, Germany; 4 Institut für Community Medicine, Universitätsmedizin Greifswald, Greifswald, Germany; 5 Heidelberg Institute for Theoretical Studies (HITS gGmbH), Schloss-Wolfsbrunnenweg 35, 69118 Heidelberg, Germany

**Keywords:** Epidemiology, Public health

## Abstract

The German initiative “National Research Data Infrastructure for Personal Health Data” (NFDI4Health) focuses on research data management in health research. It aims to foster and develop harmonized informatics standards for public health, epidemiological studies, and clinical trials, facilitating access to relevant data and metadata standards. This publication lists syntactic and semantic data standards of potential use for NFDI4Health and beyond, based on interdisciplinary meetings and workshops, mappings of study questionnaires and the NFDI4Health metadata schema, and literature search. Included are 7 syntactic, 32 semantic and 9 combined syntactic and semantic standards. In addition, 101 ISO Standards from ISO/TC 215 Health Informatics and ISO/TC 276 Biotechnology could be identified as being potentially relevant. The work emphasizes the utilization of standards for epidemiological and health research data ensuring interoperability as well as the compatibility to NFDI4Health, its use cases, and to (inter-)national efforts within these sectors. The goal is to foster collaborative and inter-sectoral work in health research and initiate a debate around the potential of using common standards.

## Introduction

The amount of health data has been growing rapidly over the past years. To search, find, (re-)use, analyze and exchange these huge amounts of data, the FAIR guiding principles – **f**indable, **a**ccessible, **i**nteroperable and **r**eusable – were established^1^. However, in healthcare as well as in health and epidemiological research, data is often not complying with any of these principles: data is frequently unstructured and stored in different, decentralized silos. This work focuses on problems related to interoperability deficiency. When there is lack of interoperability, data cannot be exchanged in a structured and meaningful manner across different institutions and software systems without substantial additional efforts.

International standards are needed to enable interoperability. Standards developing organizations (SDO) focus on the development, maintenance and promotion of standards for a specific group of users or for industry needs. The work of SDOs is mainly performed by volunteers collaborating over many years in small working groups. Proposals of each working group are usually presented to a much larger audience to achieve consensus. The number of created standards differs from one SDO to another, depending on the focus of each organization^2^. **Semantic standards** involve the use of structured vocabularies, terminologies and classification systems to represent healthcare concepts^3^. These standards ensure that health information is accurately and consistently represented across different systems, facilitating clear and precise communication within the healthcare sector. **Syntactic standards** define the structure or format of data exchange, ensuring that the meaning of data is preserved during transmission^4^. Further definitions of terms used in this manuscript are provided in **additional file 1** in our **GitHub Repository** (https://github.com/nfdi4health/IdentifiedStandards.git).

This work was performed within the NFDI4Health initiative^5^, a German research initiative developing a national research data infrastructure for personal health data. NFDI4Health represents an interdisciplinary research community which develops harmonized informatics standards for public health, epidemiological studies, and clinical trials to improve their FAIRness. We focus on the standardization of health research data to foster collaboration within these three domains. This work includes a comprehensive yet non-exhaustive list of standardization projects and initiatives at both global and national levels, along with syntactic and semantic standards. These can be utilized by the research community to describe metadata, data types, and formats from clinical, epidemiological, and public health research in a structured manner. Further standards, ontologies and terminologies might be applicable. We present an initial overview of the collaborative standardization efforts and current use of standards within a national infrastructure project for epidemiological, public health and clinical studies. Through the dissemination of these insights, we aim to empower the research community to leverage standardized practices, thereby advancing the pursuit of breakthroughs in health and medical sciences.

## Results

### Standardization efforts in health research

The independent International Organization for Standardization (ISO) is a non-governmental organization focusing on the development and publication of international standards. To date, 171 national standards bodies are members, facilitating the exchange of expert knowledge to tackle global challenges and foster innovation by developing relevant consensus-based, voluntary standards^6^. The Research Data Alliance (RDA) collects, develops and refines several standards and information to enable interoperability between research data repositories^7^. One example is the RDA COVID-19 Recommendations and Guidelines on Data Sharing^8^ that also can be seen as model for data sharing guidelines for other research studies in the health sector. In the US and Canada, the Accredited Standards Committee (ASC) is the prevailing SDO. At the European level three SDOs are responsible for defining and developing voluntary standards: the Comité Européen de Normalisation (short: CEN; for various kinds of services, processes, products and materials), Comité Européen de Normalisation Electrotechnique (short: CENELEC; for electrotechnical standardization)^9^ and European Telecommunications Standards Institute (short: ETSI; for information and communication technologies)^10^.

In the domain of healthcare, nine global initiatives work together since 2007 within the Joint Initiative Council (JIC) on solving real-world problems: Clinical Data Interchange Standards Consortium (CDISC), Digital Imaging and Communications in Medicine (DICOM), CEN/TC 251, GS1 Healthcare, Health Level 7 (HL7) International, Integrating the Healthcare Enterprise (IHE) International, ISO/Technical Committee 215, Logical Observation Identifiers Names and Codes (LOINC) and Systematized Nomenclature of Medicine (SNOMED) International. They enable real-time information exchange in healthcare by using standards based on full interoperability of information and processes^11^. The Global Alliance for Genomics & Health (GA4GH)^12^ reunites a growing number of public and private institutions from healthcare delivery and (health) research, companies, societies, funders, agencies and NGOs with the overarching goal of allowing responsible sharing of genomic data while respecting human rights. GA4GH frames policies and develops and/or refines technical standards^13^. Global Digital Health Partnership (GDHP), an international collaboration on digital health, was established in 2018 by several governments, government agencies, territories, multinational organizations and the World Health Organization (WHO). The alliance comprises currently 36 members and intercedes for the best use of digital technologies backed by evidence to improve well-being and health^14^. GDHP publishes regularly white papers about interoperability, clinical and consumer engagement, cybersecurity, policy environments and evidence and evaluation topics^15,16^. Further collaboration entail the Personal Connected Health Alliance (PCHA)^17^, or the collaboration between the American Office of the National Coordinator for Health Information Technology (ONC)^18^ and the European Union^19^ or the United Kingdom^20^. ONC serves also as the lead US representative to the GDHP^21^.

The ISO committee for standards in biotechnology (ISO/TC 276)^22^ and its working group ISO/TC 276/WG 5“Data Processing and Integration” are working on standards for data in life sciences that can and should be considered for health data (Table 3). Initial releases include guideline standards for data publication (ISO/TR 3985)^23^ and requirements for data formatting and description in life sciences (ISO 20691)^24^. Additionally, a series of standards for provenance information models for biological material and data (ISO 23494) is currently under development in ISO/TC 276/WG 5 and will be published progressively in the coming years. Moreover, in ISO/TC 215, as well as in ISO/TC 276/WG 5 several standard drafts are currently being developed for data and metadata in personalized medicine.

### Identified standards

**Fig. 1:**
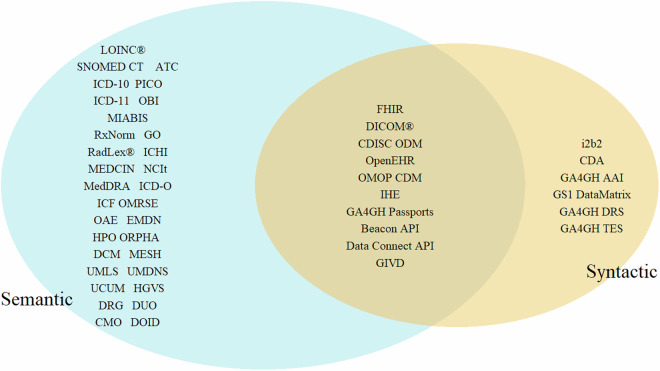
Identified syntactic and semantic standards in health research. We categorized health research and interoperability standards into three types: semantic, syntactic, or both. Semantic standards focus on the meaning and interpretation of data, including terminologies, vocabularies, and ontologies (e.g., SNOMED CT, LOINC, ICD). Syntactic standards focus on the structure and format of data exchange, defining how data is formatted and transmitted (e.g., HL7 CDA). Combined standards include elements of both, defining data structure and format while also ensuring consistent meaning with value sets or terminologies (e.g., HL7 FHIR).

We identified 7 syntactic, 32 semantic and 9 combined syntactic and semantic standards that are potentially relevant to NFDI4Health (Fig. 1). In addition, we identified further 101 ISO Standards (Table 3) from ISO/TC 215 Health Informatics and ISO/TC 276 Biotechnology, which are presented in **additional file 2**. Features of syntactic and semantic standards are represented in Table 1 and Table 2, respectively.

**Table 1:** Identified syntactic standards.

Standard	Name	Domain
FHIR	Fast Healthcare Interoperability Resources	Healthcare and research
DICOM ®	Digital Imaging and Communications in Medicine	Imaging
OMOP CDM	Observational Medical Outcomes Partnership common data model	Research (Observational research)
CDISC ODM	Clinical Data Interchange Standards Consortium Operational Data Standard	Research (Clinical Trials)
i2b2	Informatics for Integrating Biology & the Bedside	Research (Cohort discovery)
OpenEHR	—	Healthcare and research
IHE	Integrating the Healthcare Enterprise	Healthcare
CDA	Clinical Document Architecture	Healthcare
GA4GH AAI	Authentication & Authorization Infrastructure	Healthcare and research
GA4GH Passports	GA4GH Passport specification	Healthcare and research
GA4GH DRS	Data Repository Service (DRS) API	Healthcare and research
Beacon API	Beacon API	Healthcare and research
Data Connect API	Data Connect API	Healthcare and research
GA4GH TES	Task Execution Service (TES) API	Healthcare and research
GS1 DataMatrix	—	Pharmacy and Medical Devices
GIVD	Global *In Vitro* Diagnostic Product Classification	(*In vitro* diagnostic) Medical devices

**Abbreviations**: **GA4GH** – Global Alliance 4 Genomics and Health, **API** – Application Programming Interface.

**Table 2:** Identified semantic standards.

Standard	Name	Domain
FHIR	Fast Healthcare Interoperability Resources	Healthcare and research
DICOM ®	Digital Imaging and Communications in Medicine	Imaging
OMOP CDM	Observational Medical Outcomes Partnership common data model	Research (Observational research)
OpenEHR	—	Healthcare and research
IHE	Integrating the Healthcare Enterprise	Healthcare
GA4GH DRS	Data Repository Service (DRS) API	Healthcare and research
Beacon API	Beacon API	Healthcare and research
Data Connect API	Data Connect API	Healthcare and research
LOINC ®	Logical Observation Identifiers Names and Codes	Healthcare and research
SNOMED CT	Systematized Nomenclature of Medicine -Clinical Terms	Healthcare and research
ATC	Anatomical Therapeutic Chemical	Pharmacy
ORPHA	Orphanet Rare Disease Ontology	Rare Diseases
ICD-10	International Statistical Classification of Diseases, 10th Version	Health
ICD-11	International Classification of Diseases 11^th^ Revision	Health
RxNorm	—	Pharmacy
RadLex ®	—	Imaging
MEDCIN	—	EHR
NCIt	National Cancer Institute Thesaurus	Oncology
MedDRA	Medical Dictionary of Regulatory Activities	Medical products (regulation)
ICF	International classification of functioning, disability and health	Health
OAE	Ontology of Adverse Events	Health
HPO	Human Phenotype Ontology	Genetics / Phenotypes
DCM	DICOM Controlled Terminology	Radiology
MESH	Medical Subject Headings	Journal Articles
UMLS	Unified Medical Language System	Biomedical Information Services
OBI	Ontology for Biomedical Investigations	Biomedical Investigations
PICO	Patient, Population or Problem / Intervention / Comparison / Outcome Ontology	Evidence-based health
GO	Gene Ontology	Biomedical
ICHI	International Classification of Health Interventions	Health
EMDN	European Medical Device Nomenclature	Medical Devices
UMDNS	Universal Medical Device Nomenclature System	Medical Devices
MIABIS	Minimum Information About BIobank data Sharing	Biobanking
GIVD	Global *In Vitro* Diagnostic Product Classification	(*In vitro* diagnostic) Medical devices
ICD-O	International Classification of Diseases for Oncology	Oncology
UCUM	Unified Code for Units of Measure	Sciences, Engineering and Business
HGVS	Human Genome Variation Society	Genetics / Phenotypes
DRG	Diagnosis Related Groups	Medical reimbursement
DUO	GA4GH Data Use Ontology (DUO)	Healthcare and research (OMICS)
CMO	Clinical Measurement Ontology	Healthcare and research
DOID	Disease Ontology	Healthcare and research
OMRSE	Ontology of Medically Related Social Entities	Healthcare and research

**Abbreviations**: **DICOM** – Digital Imaging and Communications in Medicine, **GA4GH** – Global Alliance 4 Genomics and Health, **API** – Application Programming Interface.

### Current standards in NFDI4Health

Within NFDI4Health, a tailored metadata schema (MDS) was created to collect information from German clinical, epidemiological and public health studies collecting information on studies and their comprised study resources (e.g., study documents, instruments, data collections, etc.)^25,26^. To ensure the syntactic and semantic interoperability of the register based on the MDS, a mapping of the MDS elements to FHIR was performed and the feasibility was analyzed^27^. In addition, metadata included in the re3data^28^ schema and clinicaltrials.gov were compared to the NFDI4Health MDS. The metadata from ECRIN^29^ and DDI^30^ were also compared to the MDS. SNOMED CT, HL7 Terminology, NCIt, MeSH, ISO and ICD were used for Value Sets in the NFDI4Health MDS. The suitability of SNOMED CT for the data annotation of variables from questionnaires originating from clinical but also epidemiological and Public Health studies was evaluated by performing mappings to SNOMED CT. The results of the annotation were implemented on a test basis in OPAL/MICA^31,32^. OPAL/MICA are open software solutions built for managing and harmonizing epidemiological data^33^. With our mapping activities we evaluated suitability of different standards for our NFDI4Health use-cases.

## Discussion

Next to the existence of several SDOs responsible for developing standards in health research, we found in total, 7 syntactic, 32 semantic and 9 combined syntactic and semantic standards that may be pertinent to the epidemiological, public health and clinical research. Furthermore, we also identified an additional 101 ISO Standards sourced from ISO/TC 215 Health Informatics and ISO/TC 276 Biotechnology (Table 3).

**Table 3:** Snapshot of Identified ISO Standards. Further identified ISO Standards can be found in additional file 2.

Standard	Name	Domain
**ISO/TR 12300:2014**	Health informatics — Principles of mapping between terminological systems^42^	Health Informatics (Terminology)
**ISO 13119:2012**	Health informatics — Clinical knowledge resources — Metadata^43^	Health Informatics (Metadata)
**ISO 13940:2015**	Health informatics — System of concepts to support continuity of care^44^	Health Informatics (System of concepts to support continuity of care)
**ISO/TS 13972:2015**	Health informatics — Detailed clinical models, characteristics and processes^45^	Health Informatics (DCMs)
**ISO 14199:2015**	Health informatics — Information models — Biomedical Research Integrated Domain Group (BRIDG) Model^46^	Health Informatics (BRIDG)
**ISO 16278:2016**	Health informatics — Categorial structure for terminological systems of human anatomy^47^	Health Informatics (Terminology)
**ISO/TS 21526:2019**	Health informatics — Metadata repository requirements (MetaRep)^48^	Health Informatics (metadata)
**ISO/TS 21564:2019**	Health Informatics — Terminology resource map quality measures (MapQual)^49^	Health Informatics (Terminology)
**ISO/HL7 21731:2014**	Health informatics — HL7 version 3 — Reference information model — Release 4^50^	Health Informatics (RIM)
**ISO 27269:2021**	Health informatics — International patient summary^51^	International Patient Summary (IPS)
**ISO/HL7 27931:2009**	Data Exchange Standards — Health Level Seven Version 2.5 — An application protocol for electronic data exchange in healthcare environments^52^	Health Informatics (protocol)
**ISO/HL7 27932:2009**	Data Exchange Standards — HL7 Clinical Document Architecture, Release 2^53^	Health Informatics (clinical documents)
**ISO/HL7 27951:2009**	Health informatics — Common terminology services, release 1^54^	Health Informatics (terminology services)
**ISO/DIS 20691**	Biotechnology — Requirements for data formatting and description in the life sciences^24^	Life Sciences and Health Informatics
**ISO/DTS 23494-1**	Biotechnology — Provenance information model for biological material and data — Part 1: Design concepts and general requirements^55^	Life Sciences, especially biobanking and data processing, as well as health informatics
**ISO/TR 3985:2021**	Biotechnology — Data publication — Preliminary considerations and concepts^56^	Life Sciences and Health Informatics
**ISO/AWI TS 6201**	Health informatics — Personalized Digital Health – Framework^57^	Health Informatics (Personalized digital health)
**ISO/PWI TS 6203**	Health informatics — Personalized digital health – Standard data set for frailty assessment^58^	Health Informatics (Personalized digital health)
**ISO/AWI TR 11147**	Health informatics – Personalized digital health – Digital therapeutics health software systems^59^	Health Informatics (Personalized digital health)
**ISO/CD 9472-10000**	Health informatics — Personalized health navigation – Architecture^60^	Health Informatics (Personalized digital health)
**ISO/AWI TR 24305**	Health informatics — Guidelines for implementation of HL7/FHIR based on ISO 13940 and ISO 13606^61^	Health Informatics (Interoperability, FHIR)
**ISO/PRF 13972:2022**	Health informatics — Clinical information models — Characteristics, structures and requirements^45^	Health Informatics (DCMs)
**ISO/CD 29585**	Health informatics – Framework for healthcare and related data reporting^62^	Health Informatics
**ISO/HL7 10781:2023**	Health Informatics — HL7 Electronic Health Records-System Functional Model, Release 2 (EHR FM)^63^	Health Informatics (Interoperability, FHIR)
**ISO/DIS 4454**	Genomics informatics — Phenopackets: A format for phenotypic data exchange^64^	Health Informatics

While there is literature and guidance on the use of standards in health records^34^, our literature review revealed a notable lack of comprehensive overviews to guide the selection of standards for health research studies.

In our use case for NFDI4Health, we specifically focused on metadata describing studies and study questionnaires. Our ultimate goal is to make these metadata elements exchangeable and comparable across clinical, epidemiological, and public health studies.

The World Health Organization’s “International Standards for Clinical Registries” does not specifically recommend any semantic or syntactic standards. It merely advises that “in addition to free text, controlled vocabularies may be used,” citing SNOMED, ICD, and MeSH as examples and recommending controlled vocabularies that can be mapped to the Unified Medical Language System (UMLS) Metathesaurus, as used by the ICTRP Search Portal^35^. In our current MDS, we implemented SNOMED and MeSH amongst NCI, LOINC and ISO and provided concept maps to UMLS^36,37^.

The “Second Joint Action Towards the European Health Data Space – TEHDAS2” project provided a list of relevant standards for harmonizing semantic and syntactic interoperability in the European Health Data Space^38^. Our list includes all the semantic and syntactic standards identified by this working group. However, they also provided a list of metadata standards, which we did not explore in this manuscript. When developing the MDS, we performed mappings to several of these metadata standards, such as ECRIN which we will report on the future.

Harmonization of retrospective data is only one goal of NFDI4Health. There is a need to increase global awareness about the importance of standards and to incentivize the prospective use of internationally, widely recognized standards in studies, starting with the planning phase. As NFDI4Health targets health data, it is crucial to apply standards used in the healthcare system such as SNOMED CT, ICD and LOINC. By doing so, the entire community may benefit from improved data exchange possibilities.

Due to the heterogeneity of studies, multiple standards might need to be combined based on the specific needs and variables assessed. Evaluating the mappings between these standards is also essential. However, it is important to avoid creating further data silos by installing an excessive number of standards, which could hinder interoperability. Relying on existing concepts and aligning with other projects can benefit the entire community by improving data exchange possibilities. This vast array of health standards highlights the need for interoperability at an organizational level to implement standards on a consensus basis. Therefore, it is essential to consider already existing guidelines and established standards on both national and international levels. For NFDI4Health, this means considering already established data models and standards in the German healthcare system as well as other projects, such as the medical informatics initiative^39^. The transport and content standard HL7 FHIR is part of several new requirements in the European healthcare system to rely on one common standard^40^. FHIR is easy to use, adaptable and relies on already existing web technologies which can be used in web and mobile applications, and we therefore decided to use it as exchange standard for our MDS^36^. Of course, other standards are not to be missed and will be identified according to the requirements of the use cases. This work serves as basis for future (meta-)data repositories, establishing services necessary to harmonize and standardize (meta-)data, enabling analyze and access those (meta-)data, and introducing relevant guidelines for the entire NFDI4Health consortium and beyond.

## Methods

### Identification of standards

To identify relevant SDOs and standards for health(care) data within NFDI4Health, we conducted a literature search and searches in community-driven portals such as FAIRsharing, BioPortal, and EMBL-EBI Ontology Lookup Service (short: OLS Ontology Search) as well as the website of ISO. In addition, over a time of three years, we gathered information from use cases and domain experts in the field of health research and interoperability. Therefore, interviews were conducted with each of the five use cases at least once and up to three times. We held a workshop on metadata standards with the entire community discussing the community needs and identifying relevant standards. We performed mappings of study instruments and the developed metadata schema to international standards and analysed these for their suitability and finally developed value sets to be used in NFDI4Health’ MDS^27,32^. Standards such as terminologies, ontologies, vocabularies were considered semantic standards. We included requirements from the user community and their use cases, feedback and experiences, existing guidelines and recommended (inter-)national standards. Each activity was reviewed in interdisciplinary biweekly meetings and commented by the general assembly of NFDI4Health. All authors have either significant expertise and/or practical experience in the field of interoperability and/or health research.

### Categorization of standards

In this study, we categorized standards used in health research into three categories: semantic, syntactic, or both. The categorization process was based on specific criteria related to the nature and application of each standard. Standards were classified as semantic if they primarily focused on meaning and interpretation of data. This included terminologies, vocabularies, and ontologies. Examples of such standards include SNOMED CT, LOINC, and ICD. These standards provide a structured way to describe the data and ensure consistent interpretation across different systems and contexts. Standards were classified as syntactic if they focused on the structure and format of data exchange. These standards define how data is formatted, encoded, and transmitted between systems. Examples include HL7 CDA. These standards ensure that the data can be correctly parsed and understood at a structural level by receiving systems. Some standards encompass both semantic and syntactic elements. These standards not only define the structure and format of data but also include value sets or terminologies for ensuring consistent meaning. An example of such a standard is HL7 FHIR, which includes both a syntactic framework for data exchange and its own value sets for semantic consistency.

### Analysis

Results were presented in tables and Fig. 1 was created using R statistical software (version 2024.04.1; R Foundation for Statistical Computing)^41^ and the VennDiagramm packages.

## Data Availability

All identified standards can be found in our GitHub repository (https://github.com/nfdi4health/IdentifiedStandards.git)

## Code availability

The code for generating Fig. 1 is provided in our GitHub repository (https://github.com/nfdi4health/IdentifiedStandards.git).
